# 

**Published:** 1878-08

**Authors:** 


### Books and Pamphlets Received.

---

Transactions of the American Gynecological Society, Vol. II, for the year 1877. Boston: Houghton, Osgood & Co. 1878. Buffalo: Peter Baul & Bro.

Report of the Commissioners of Education, for the year 1876. Washington: Government Printing Office. 1878·

Nervous Diseases. Their Description and Treatment, by Allan McLane, M. D. Philadelphia; Henry C. Lea. 1878. Buffalo: Theodore H. Butler.

Visions, study of false sight, by Edward H. Clark, M. D. Boston: Houghton, Osgood & Co. 1878. Buffalo: Peter Paul & Bro.

A Manual of Operative Surgery, by Lewis A. Stimson, M. D. Philadelphia: H. C. Lea. 1878. Buffalo: T. H. Butler.

Litholapaxy, or rapid Lithotrity with Evacuation, by Henry J. Bigelow, M. D. Boston: A. Williams & Co. New York: Wm. Wood & Co. 1878.

Antagonism of Alcohol and Diphtheria, by E. U. Chapman, A. M. M. D. Brooklyn Union Argus Printing Establishment. 1878.

How to take care of our Eyes, by Henry C. Angell, M. D. Boston: Roberts Brothers. 1878. Buffalo: Peter Paul & Bro.

Atlas of Skin Diseases, Part IV, by Louis A. Duhring, M. D. Philadelphia: J. B. Lippincott & Co. 1878.

American Journal of Obstetrics and Diseases of Women and Children. July, 1878.

American Journal of Insanity. July, 1878.

American Journal of the Medical Sciences. July, 1878.

Economy and Necessity of a State Board of Health, by L. D. Waterman, M. D. Indianapolis. 1878.

Remarks on Ovariotomy, by J. W. Rosebrugh, M. D.

Certain Symptoms of Nervous Exhaustion, by George M. Beard, M. D. Reprint from Virginia Medical Monthly. June, 1878.

A Case of Vaginal Ovariotomy, by Wm. Goodell, A. M. M. D. Reprint from Vol. I, Gyneological Transactions. 1878.

Remarks on Social Conservatism, by J. W. Singleton, M. D.

Address before the Tioga Medical Society, by J. W. Singleton, M. D.

The Obstetrical Forceps, When and How to Use it, by Geo. J. Englemann, M. D.

The Hystero-Neuroses, by Geo. J. Englemann, M. D. Reprint from Vol. II, Gynecological Transactions. 1878.

A Hystero-Psychosis, by Geo. J. Englemann, M. D. St. Louis. 1878.

Medical Register for Albany and other Counties for 1878–9.

Application of Pressure in Diseases of the Uterus, by V. H. Taliafeiro, M. D., Atlanta.

The Functions of the Anal Sphincters, so called, by James R. Chadwick, M. D.

Cases of Double Uterus and Vagina, by James R. Chadwick, M. D.

The Soft Palate, its value in Diagnosis, by Wm. A. Love, M. D.

Amputations and Excisions of the Cervix Uteri; their Indications and Methods, by J. Byrne, M. D., R. C., S. E.

Vascular Tumors of the Female Urethra, by A. Reeves Jackson, A. M., M. D.

Old Age, its diseases and Hygiene, by L. P. Yandell, M. D.

Eulogy upon Lunsford P. Yandell, M. D., by T. S. Bell, M, D.

New Treatment for Spine Diseases, by Meigs Case, M. D.

S.G.O. INDEXED

400

### THE BUFFALO MEDICAL AND SURGICAL JOURNAL

MEDICAL AND SURGICAL JOURNAL.

PUBLISHED MONTHLY,—Containing Original Articles, Reports of Medical Societies and Hospitals, Editorial, Reviews, Correspondence, Army News, etc., etc; including the usual variety of Medical Periodical Publications. Specimen copies sent on application to BUFFALO MEDICAL AND SURGICAL JOURNAL, J. F. MINER, M. D., 978 Main Street, BUFFALO, N. Y.

TERMS OF SUBSCRIPTION, $3.00 PER YEAR, IN ADVANCE,—All communications for publication should be sent before the fifteenth (15) of each month, or they will of necessity lie over until the next number. No change can be made in advertisements after the twenty-fifth.

Correspondence and original articles are solicited. Publication is no evidence that the views of the author are endorsed by the Journal.

